# Supplementary material for: Electrocatalytic valorization of lignocellulose-derived aromatics at industrial-scale current densities
Source: Nat Commun. 2023 Nov 9;14:7229. doi: 10.1038/s41467-023-43136-y (PMC10636212; doi:10.1038/s41467-023-43136-y)
Supplement: Supplementary file 1 — Supplementary Information [file 41467_2023_43136_MOESM1_ESM.pdf]

Supporting information

Electrocatalytic valorization of lignocellulose-derived aromatics at industrial-scale current densities

Tao Peng[†], Wenbin Zhang[†], Baiyao Liang, Guanwu Lian, Yun Zhang, Wei Zhao*

Institute for Advanced Study, Shenzhen University, Shenzhen, Guangdong 518060, China.

[†] These authors contributed equally

[*] Corresponding author Email: weizhao@szu.edu.cn

This PDF file includes:

Supplementary Experimental Section, Techno-economic analysis (TEA)

Supplementary Tables 1 to 2

Supplementary Figures 1 to 17

Supplementary Experimental Section

Chemical and materials

Nafion[tm] 117 membrane (Fuel Cell Store), carbon felt (Avcarb G300a, Fuel Cell Store) nickel foam(80-120 PPI - 1 mm, Fuel Cell Store), copper foam (1.6mm thickness, MTI Corp); perchloric acid ($HClO_4$, 70%, Innochem), ethanol (99.7%, Innochem), sodium sulfate (Na_2SO_4, 99%, Innochem), rhodium chloride hydrate ($RhCl_3$, Innochem), irridium chloride hydrate ($IrCl_3$), Palladium (II) chloride ($PdCl_2$, Innochem), chloroplatinic acid (H_2PtCl_6, Innochem), furfuryl alcohol (FA, 98%, Innochem), tetrahydrofurfuryl alcohol (THFA, 99%, Innochem), Guaiacol (99%, Acros Organics), 4-Propylcyclohexanone (4PCN, 99%, Sigma-Aldrich), Syringol (2,6-dimethoxyphenol, 99%, Innochem), 2-methoxycyclohexanol (2MCHol, 98%, Alfa Aesar), 2-methoxycyclohexanone (2MCHN, 98%, Elite Chemie), cyclohexanol (CHol, 99%, Innochem), cyclohexanone (CHN, 99%, Alfa Aesar).

Methods

Electrochemical properties. Electrochemically active surface areas (ECSAs) were determined by Cu underpotential deposition (Cu_{UPD}) stripping method.[1] Briefly, CVs on catalysts in N_2-purged 0.1 M H_2SO_4 solution were first performed at a scan rate of 10 mV/s to obtain stable CVs as the background. Then, Cu_{UPD} was performed at a scan rate of 10 mV/s in a N_2-purged 0.1 M H_2SO_4 solution containing 2 mM $CuSO_4$ at 25℃ after Cu deposition at 0.30 V (vs. RHE) for 100 s (**Figure S14**). Hydrogen underpotential deposition (H_{UPD}) experiment was conducted at a scan rate of 10 mV s^{-1} in an N_2-saturated 0.5 M H_2SO_4 solution. Take the average value of the coulombic charge in the shaded region in **Figure S15**, and divide it by the surface charge density (Q_s: 221 μC cm^{-2})[2], to obtain the ECSA of the Rh catalyst.[3] The ECSA obtained from the Cu_{UPD} test was 5.073 m^2/g (or 314 cm^2 for 6.2 mg Rh catalyst), while H_{UPD} test delivered a result of 5.23 m^2/g.

ECSA (m^2/g_{metal}) values was calculated using equation:

$$Q_{\mathrm{Cu}} = \frac{I*V}{\text{scan rate}} \tag{1}$$

$$\mathrm{ECSA} = \frac{Q_{Cu}}{\mathrm{Q}_S \times m_{metal}} \tag{2}$$

$$N_{\mathrm{Rh}} = N_{\mathrm{Cu}} = Q_{\mathrm{Cu}}/2\mathrm{F} \tag{3}$$

$$Q_{\mathrm{H}} = \frac{I*V}{\text{scan rate}} \tag{4}$$

$$\mathrm{ECSA} = \frac{Q_H}{\mathrm{Q}_S \times m_{metal}} \tag{5}$$

Where Q_{Cu} is the measured integral charge ($Cu_{UPD} \rightarrow Cu^{2+} + 2e^{-}$), Q_s is the surface charge density of 4.07 C/m^2 with a assumption of a monolayer adsorption of Cu_{UPD} on Rh, m_{metal} is the Rh mass (6.2 mg); N_{Rh} is the moles of surface Rh.

ECH product analysis.

The liquid products were collected at one hour and other specific times as indicated in the main text. The liquid samples were qualitatively analyzed using a GC-MS (PerkinElmer Clarus 680) equipped with a Stabilwax column (fused silica, Restek). The liquid products were quantified using a GC (Agilent 8890) equipped with a DB-WAX UI column (Agilent). An FID detector was used as detector; 4-propyl-cyclohexanone was used as the internal standard. Calibration was performed using the internal standard method, and a standard curve of the product was prepared for quantitative analysis of the experimental results. Injector temperature was set to 250°C with a split ratio of 30:1. The oven program started at 80°C for 1 min, heated to 140°C at 60°C/min and then to 190°C at 50°C/min, and then to 210°C at 40°C/min and the temperature was held for more than 1 min. The current density (J) was determined based on geometric area in this study. The FE toward products was calculated using the equation:

$$\mathrm{FE} = \frac{e \times n \times \mathrm{F}}{I \times t} \tag{6}$$

where e is the number of electrons transferred in this product, n (in moles) is the amount of a specific product, F is the faradaic constant, I (in amperes) is the current, and t (in seconds) is the time for the continuous current. We further performed bulk ECH of guaiacol using our engineered flow-cell system with 1 L of electrolyte circulated using a peristaltic pump over 32 hours of a continuous reaction.

The conversion rate (C%) and yield (Y%) of bio-oil compound were calculated using equations:

$$C\% = \frac{\text{moles of bio−oil compund consumed}}{\text{initial moles of bio−oil compund}} \times 100\% \tag{7}$$

$$\mathrm{Y}\% = \frac{moles\ of\ target\ product}{\text{initial moles of bio−oil compound}} \times 100\% \tag{8}$$

The turnover frequency (TOF, h^{-1}) for production of ECH target product is calculated using equation:

$$\mathrm{TOF} = \frac{N_{\mathrm{P}}}{\mathrm{N_{Rh}} \times t} \tag{9}$$

$$J = \frac{I}{A} \tag{10}$$

$$\mathrm{FE} = \frac{N_P * Z_P * \mathrm{F}}{I * t} \times 100\% \tag{11}$$

$$J_{\mathrm{p}} = J * \mathrm{FE} \tag{12}$$

$$\frac{N_P}{t} = \mathrm{FE} * \frac{I}{\mathrm{Z_P * F}} = \mathrm{FE} * \frac{J * A}{\mathrm{Z_P * F}} = \frac{J_p * A}{Z_P * F} \tag{13}$$

$$\mathrm{TOF} = \frac{N_\mathrm{P}}{\mathrm{N_{Rh}} \times t} = \frac{J_p * A}{\mathrm{N_{Rh}} * \mathrm{Z_P} * \mathrm{F}} = \frac{A}{\mathrm{Z_P} * \mathrm{F}} * \frac{J_\mathrm{P}}{\mathrm{N_{Rh}}} \tag{14}$$

Where N_p is the moles of total target products formed; N_{Rh} is the moles of surface Rh atoms; t is the time duration for production of N_p moles target products; F is the faradaic constant; J is total current density; I is total current; A is geometric area of work electrode; Z_P is the number of electrons transferred for one molecule of target product formed;

Productivity, FE and Current density

The productivity is proportional to partial current density, which is the FE to target product multiplying the total current density. See the following deduction process:

$$J = \frac{I}{A} \tag{15}$$

$$\mathrm{FE} = \frac{n_i * Z_i * F}{I * t} \times 100\% \tag{16}$$

$$\text{so, } \frac{n_i}{t} = FE * \frac{I}{Z_i * F} \tag{17}$$

$$\text{productivity} = \frac{n_i}{A * t} \tag{18}$$

$$\text{so, productivity} * A = \frac{n_i}{t} = FE * \frac{I}{Z_i * F} \tag{19}$$

$$\text{thus, productivity} == \frac{1}{Z_i * F} * FE * j = \mathrm{C} * FE * j \tag{20}$$

where, j is total current density; I is total current; A is area of catalyst; n_i is the number of mole of product i formed; Z_i is the number of electrons transferred for one molecule i formed; F is the Faraday constant (96485.333 C/mol); t is the ECH reaction time; C $(= \frac{1}{Z_i * F})$ is constant.

Materials characterization. The morphology of cathode was investigated using HRTEM and scanning transmission electron microscopy (STEM) (JEM-2100F/ FEI Talos F200S) equipped with EDX detectors. XPS analysis of Rh catalyst was carried out on PHI Genesis 900 with Al Kα.

***In-situ* infrared reflection-absorption spectroscopy (IRRAS).** IRRAS measurements were performed using a Nicolet iS50 spectrometer equipped with an A-type mercuric cadmium telluride (MCT) detector.[5] The working electrode was against the flat surface of a ZnSe hemisphere.

Table S1. Selected prior reports for electrocatalytic biorefinery of bio-oil aromatics. Abbreviations: Current density (J); faradaic efficiency (FE); partial current density (J_p); KA oil is the mixture of cyclohexanone ketone and cyclohexanol alcohol.

**System**	Catalyst	**T (°C)**	**Bio-oil compound**	**Product**	**J mA/cm²**	**FE %**	**J_p mA/cm²**	**Loading mg/cm²**	**Potential vs. RHE (V)**	**Reference**
**MEA**	**Rh/CF**	**25**	**Guaiacol**	**Methoxy-cyclohexane Pharmaceuticals**	**100**	**68**	**68**	**6.2**	**-0.10**	**This work**
**MEA**	**Rh/CF**	**25**	**Furfural alcohol**	**Tetrahydro furfuryl alcohol**	**200**	**67**	**134**	**6.2**	**-0.30**	**This work**
**MEA**	**Rh/CF**	**25**	**Guaiacol**	**Methoxy-cyclohexane Pharmaceuticals**	**300**	**64**	**192**	**6.2**	**-0.58**	**This work**
Improved H-cell	Rh/CF powders	18	Methoxyphenol	4-Methoxycyclohexanone mixture	21	35	7.35	0.2	-0.4	*J. Catal., 2016, 344 263-272.*[4]
Flow cell	PtNiB	60	Guaiacol	KA oils	5	80 ~ 90	4 ~ 4.5	2	*-0.21*	*Adv. Funct. Mater. 2019, 29, 1807651.*[6]
H-cell	Pt/C	50	Furfural	Furfural alcohol	30	85	25.5	*1*	*-0.25*	*Electrochim. Acta. 2014, 135 139–146.*[7]
PEM fuel cell	Pd/C	25	Furfural	Tetrahydrofurfuryl alcohol	19	25	4.8	*1*	*-0.22*	*Green Chem. 2013, 15, 1869-1879.*[8]
H-cell	Raney Ni	75	Mequinol	4-Methoxycyclohexanol	8	26	2.1	*12*		*Green Chem. 2015, 17, 601-609.*[9]
H-cell	Ru	50	Guaiacol	Methoxy-cyclohexanes	25	19	4.8			*Green Chem. 2012, 14, 2540-2549.*[10]
H-cell	Ru	80	Syringol	Methoxy-cyclohexanes	25	49	12.3			*Green Chem. 2012, 14, 2540-2549.*[10]
H-cell	Ru/ACC	80	Guaiacol	2-methoxycyclohexanol	100	30	30			*Green Chem., 2012, 14, 2540–2549.*[10]
H-cell	Ru	80	Guaiacol	Cyclohexanol	22	45	9.9			*ACS Sustain. Chem. Eng. 2019, 7, 8375-8386.*[11]
H-cell+ slurry reactor	Pt/C	50	Guaiacol	2-methoxycyclohexanone	109	72	78.5	*9.5*	*-1.7*	*J. Appl. Electrochem. 2021, 5151-63.*[12]
H-cell+ slurry reactor	Pt/C	60	Guaiacol	Cyclohexanone	146	35	51	*2.55*	*-2.1*	*Green Chem. 2022, 24, 7469-7480.*[13]

H-cell+ slurry reactor	Pt/C	50	Guaiacol	2-methoxycyclohexanol	109	94	102	*2.4*	*-1.72*	*ChemSusChem 2020, 13, 629-639.*[14]
H-cell+ slurry reactor	Pt/C	24	Guaiacol	Cyclohexanol	150	75	112.5	*1.8*	*-1.1*	*ACS Sustainable Chem. Eng. 2021, 9, 13164-13175.*[15]
Flow cell	Pd	25	Benzaldehyde	Benzyl alcohol	15	52	7.8	*0.25*	*-0.49*	*ACS Sustain. Chem. Eng. 2020, 8, 4407-4418.*[16]

Techno-economic analysis (TEA)

We performed a TEA to evaluate the technoeconomic feasibility of ECH of bio-oil compounds using a modified model (**Table S2**) of previous works [17,18]. The plant-gate levelized costs of various products (unit price US$ per kg or ton of products) were calculated using the following assumptions: **1.** The plant capacity is fixed at 10 tons/day to produce THFA from FA and KA-oil from guaiacol and 100 kg/day to produce high-value 2MCHol from guaiacol/syringol; **2.** The catalyst cost includes Rh cathode cost, other catalyst cost (10% Rh cost), and synthesis cost (5% all catalyst costs). **3.** The membrane cost is 5% of the total electrolyzer cost ($1840/m^2 of the base case as reported[19]); **4.** The price of renewable electricity is 7.6 ¢/kWh that is the average price of bioenergy in 2020; **5.** Extractive distillation is assumed to separate the products with a cost of ¢14.5/kg for methoxy-cyclohexanes/ cyclohexanes and $40.9/ton for THFA that is larger than the reported maximum cost for separation of cyclohexane-based products [20]; **6.** Other operation cost is assumed to be 10% of the electricity cost; **7.** Assume a plant capacity factor of 0.67, indicating an operational hour of 16 h/day; **8.** The prices of guaiacol, syringol, and 2MCHol are $5/kg, $30/kg, and $430/kg, respectively, and the prices KA-oil, FA, and THFA are $1600/ton, $1200/ton and $3500/ton, respectively. **9.** In **Figure S1** for KA-oil (the mixture of cyclohexanol and cyclohexanone with a molar ratio of 1:1) from ECH of guaiacol by use of Pt cathode, the electron transfer number and product molecular weight are assumed to be the average electron transfer number and average molecular weight; at various current densities the FEs are assumed to be 80% and other calculated data are assumed to be similar as the TEA analysis of methoxy-cyclohexanes from ECH of guaiacol by utilization of Rh cathode.

Table S2. Model of techno-economic analysis (TEA) for ECH of bio-oil compounds. Modified model from the previous works.[17,18]

Breakdown of TEA	Details
**Capital cost**	Electrolyzer ($1840/m^2) cost + catalyst cost + membrane cost (5% of the electrolyzer) + electrolyte cost
**Installation cost**	Lang factor (50%)×(electrolyzer cost + catalyst cost + membrane cost)
**Maintenance cost**	Maintenance frequency (1/day) × maintenance factor (5%) × (electrolyzer cost + catalyst cost + membrane cost)
**Balance of plant**	Balance of plant factor (35%)×(electrolyzer cost + catalyst cost + membrane cost)
**Operational cost**	Product separation cost (40.9 $/ton of the THFA from furfural alcohol ECH, and 145 $/ton of the KA oil from guaiacol ECH, 2MC from guaiacol ECH, and 2MC from syringol ECH) + other operational cost (10% of the electricity cost)
**Electricity cost**	Full-cell potential, FE, and electricity price
**Input chemical cost**	Bio-oil compound cost + Electrolyte cost ($HClO_4$ cost + Water cost)

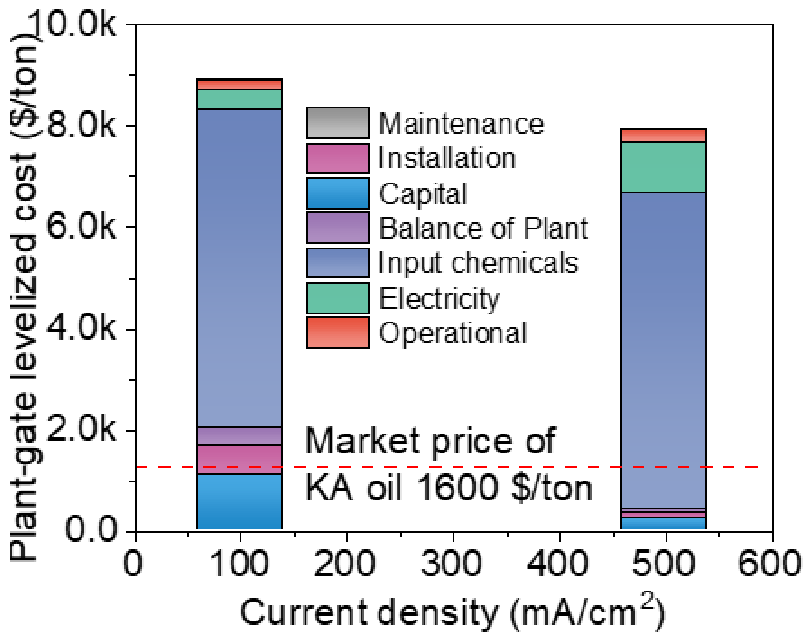

Figure S1. Breakdown of TEA costs of KA oil from ECH of lignin monomer guaiacol at 100 and 500 mA cm^{-2}. We assumed a high FE of 80% to KA oil using a flow cell and Pt cathode, showing that costs of KA oil from ECH of guaiacol far exceeds the market price of $1600/ton.

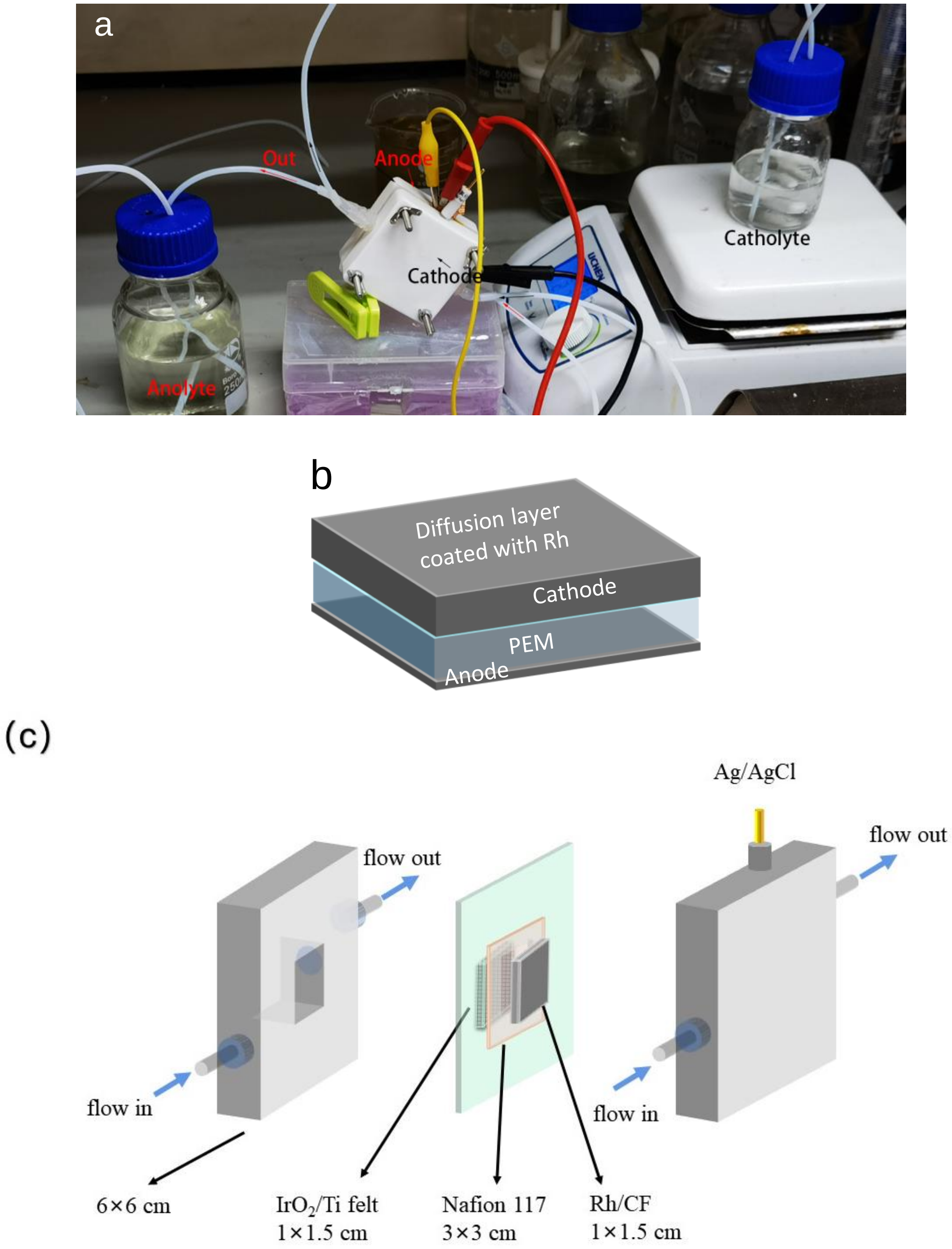

Figure S2. (a) The electrolyte was circulated through the flow cell. (b) Schematic illustrating our custom-made membrane electrode assembles (MEA) consisting of a catalyst coated diffusion layer, proton exchange membrane (PEM) and IrO_2/Ti mesh anode. These three layers are closely packed to minimize the Ohmic resistance and electrode potentials. (c) Scheme of MEA flow-cell with the detailed parameters.

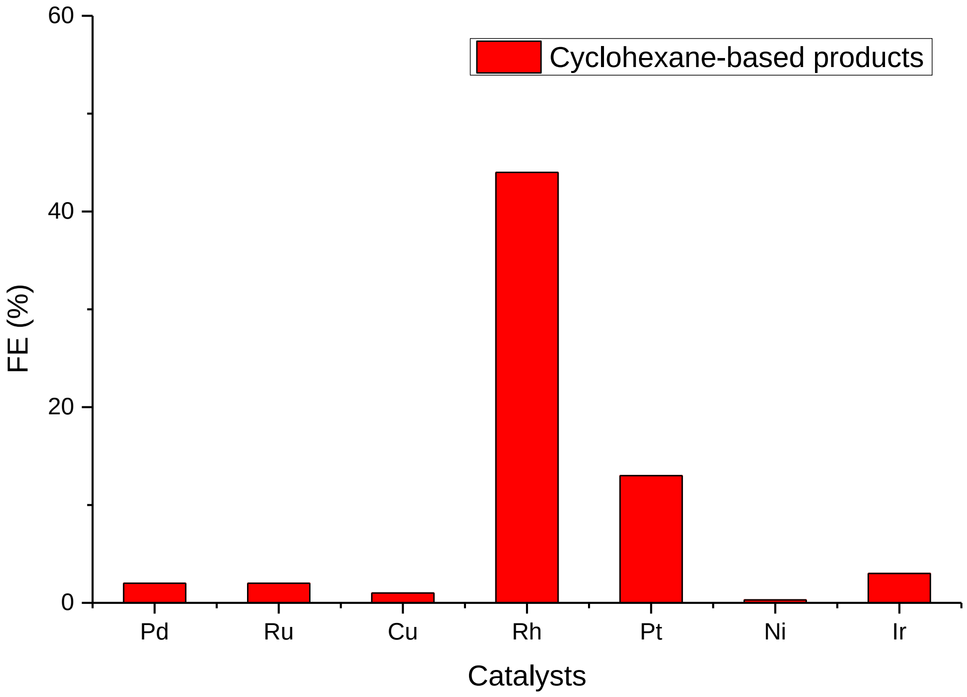

Figure S3. Catalyst screening using the ECH of guaiacol at applied current density of 300 mA/cm^2 using a H-cell system. The cyclohexane-based products include 2MCHol, 2MCHN, CHol, and CHN. This figure indicates the Rh shows good FE to catalyze the hydrogenation of guaiacol to cyclohexane-based products.

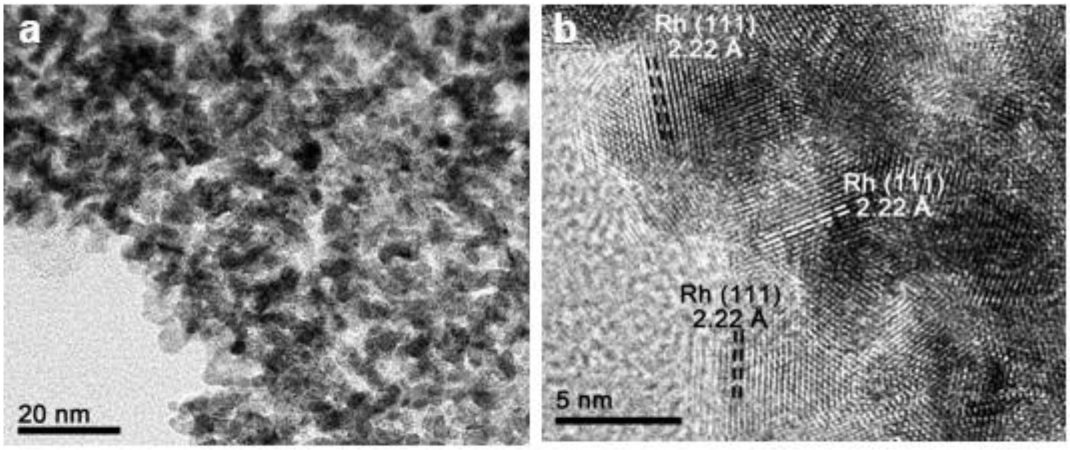

Figure S4. (a, b) TEM (a) and HRTEM (b) images of Rh nanopartilces from the carbon felt. These Rh nanoparticles with a particle diameter of ~5nm, showing the Rh (111) crystal interplanar spacing of 2.22 Å.

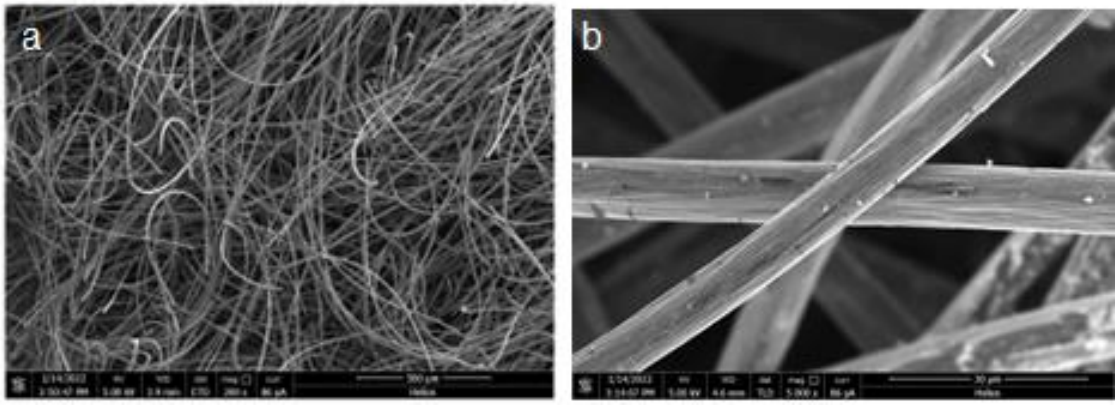

Figure S5. (a-b) SEM image of Rh coated on the carbon felt.

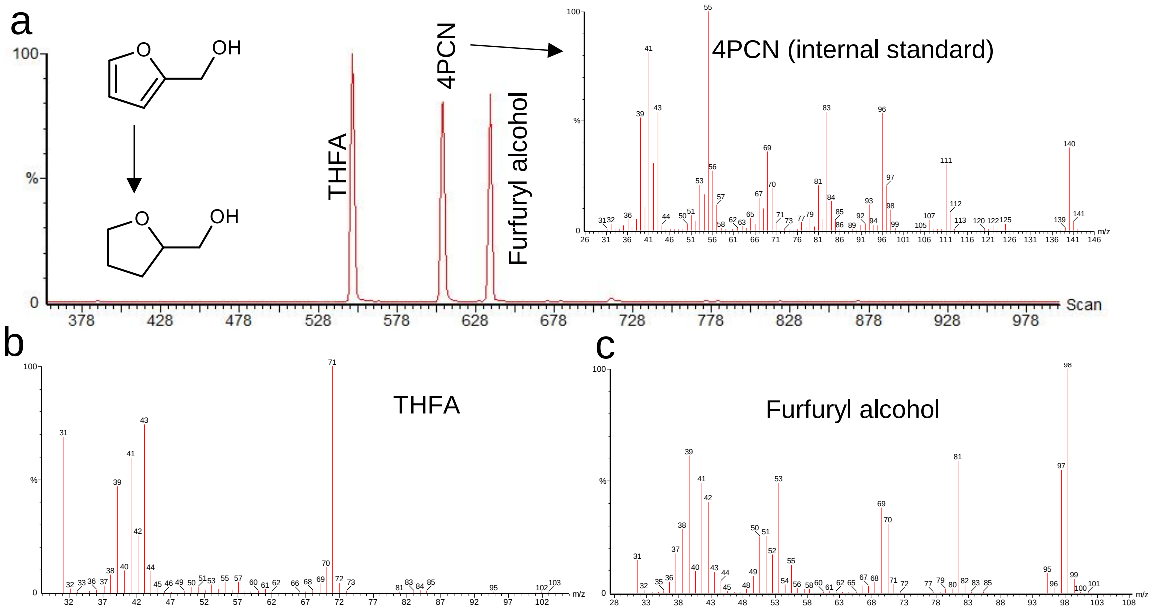

Figure S6. GC-MS results of THFA from ECH of FA on Rh/CF catalyst at 100 mA/cm^2 after a course of 1-h reaction. (a) GC-MS analysis. (b) Mass spectra from GC-MS analysis of THFA. (c) Mass spectra from GC-MS analysis of furfuryl alcohol.

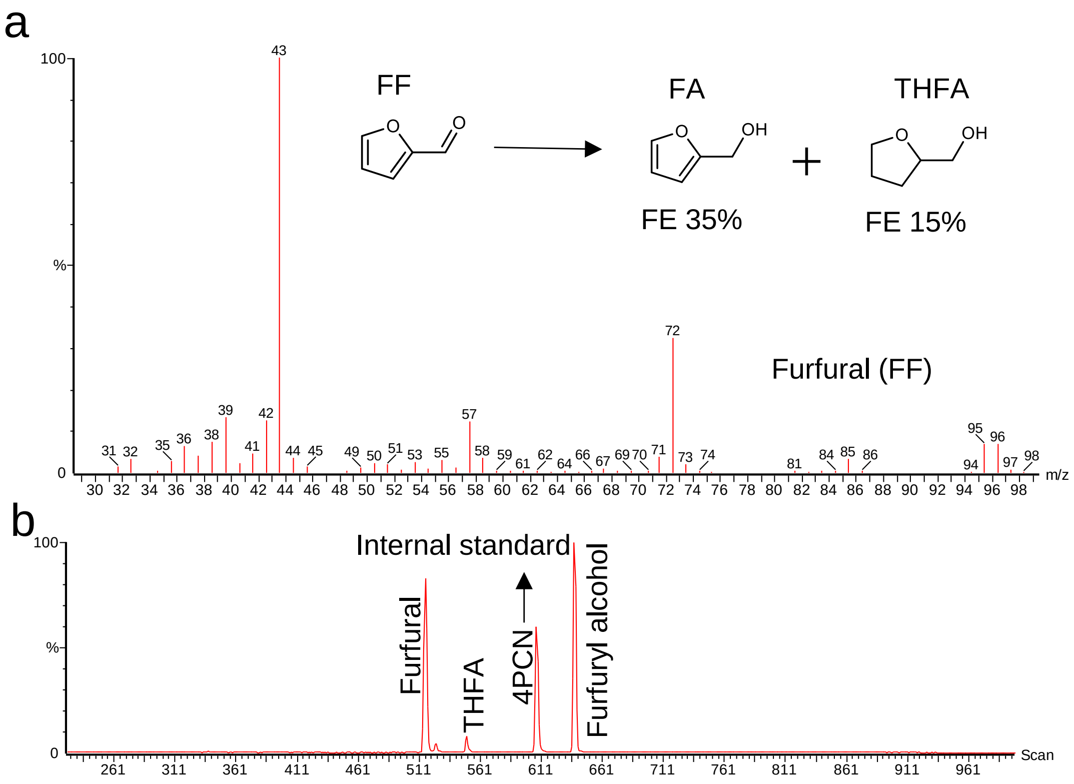

Figure S7. GC-MS results of FA and THFA from ECH of FF on Rh/CF catalyst at 200 mA/cm^2 after a course of 1-h reaction. The FF was hydrogenated to FA and THFA with total FE of 50% in a course of 1-h ECH. The FA was eventually converted to THFA with FE of 41% in a course of 2-h ECH. The FE to FA from FF is 14% in a course of 2-h ECH. The FA peaks was determined in Figure S6. (a) Mass spectra from GC-MS analysis of FF. (b) GC-MS analysis of ECH reaction.

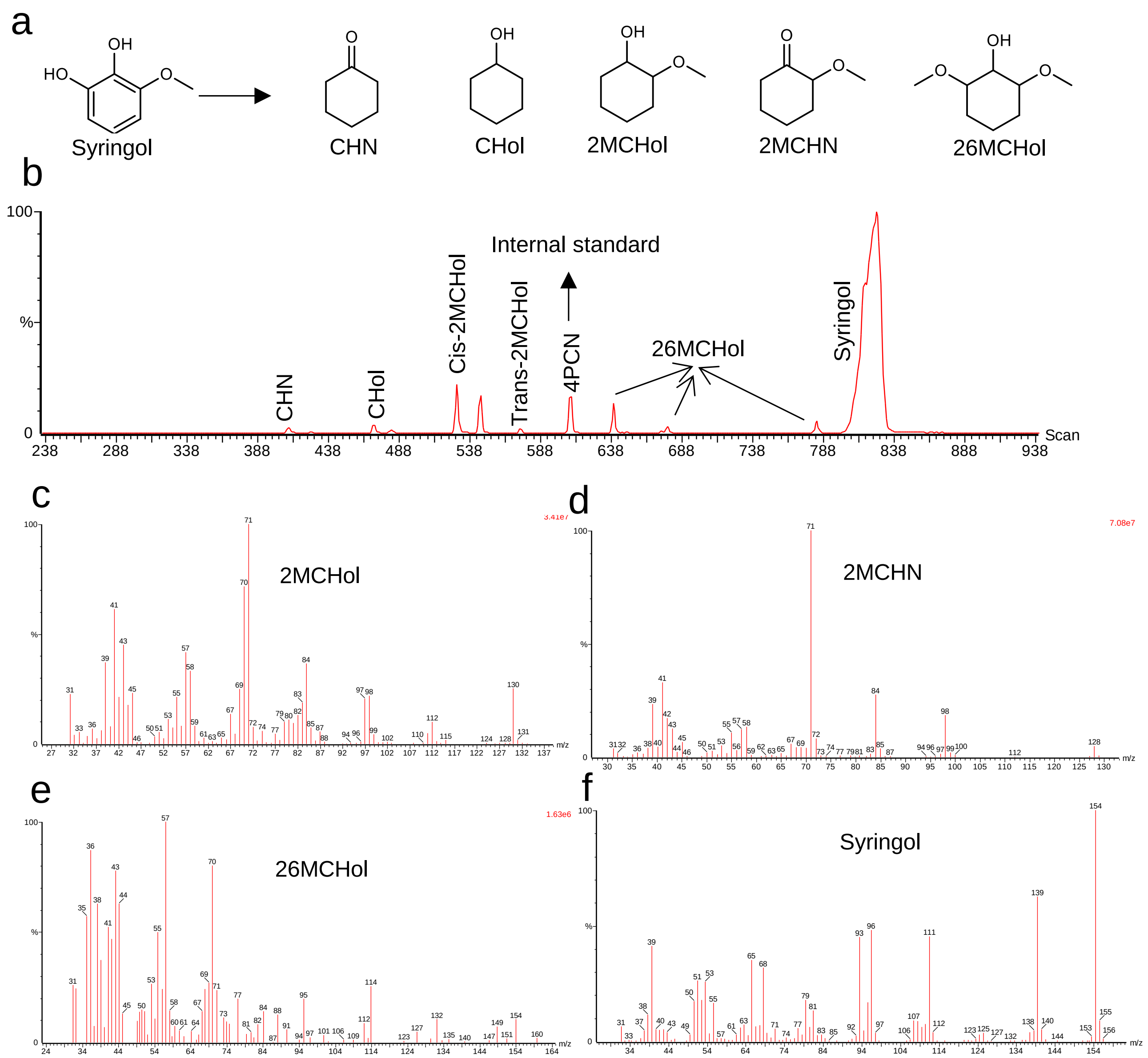

Figure S8. GC-MS results of products from ECH of syringol on Rh/CF catalyst. (a) ECH of syringol to CHN, CHol, 2MCHol, 2MCHN, and 26MCHol. (b) GC-MS analysis of ECH reaction. (c) Mass spectra from GC-MS analysis of 2MCHol. (d) Mass spectra from GC-MS analysis of 2MCHN. (e) Mass spectra from GC-MS analysis of 26MCHol. (f) Mass spectra from GC-MS analysis of syringol.

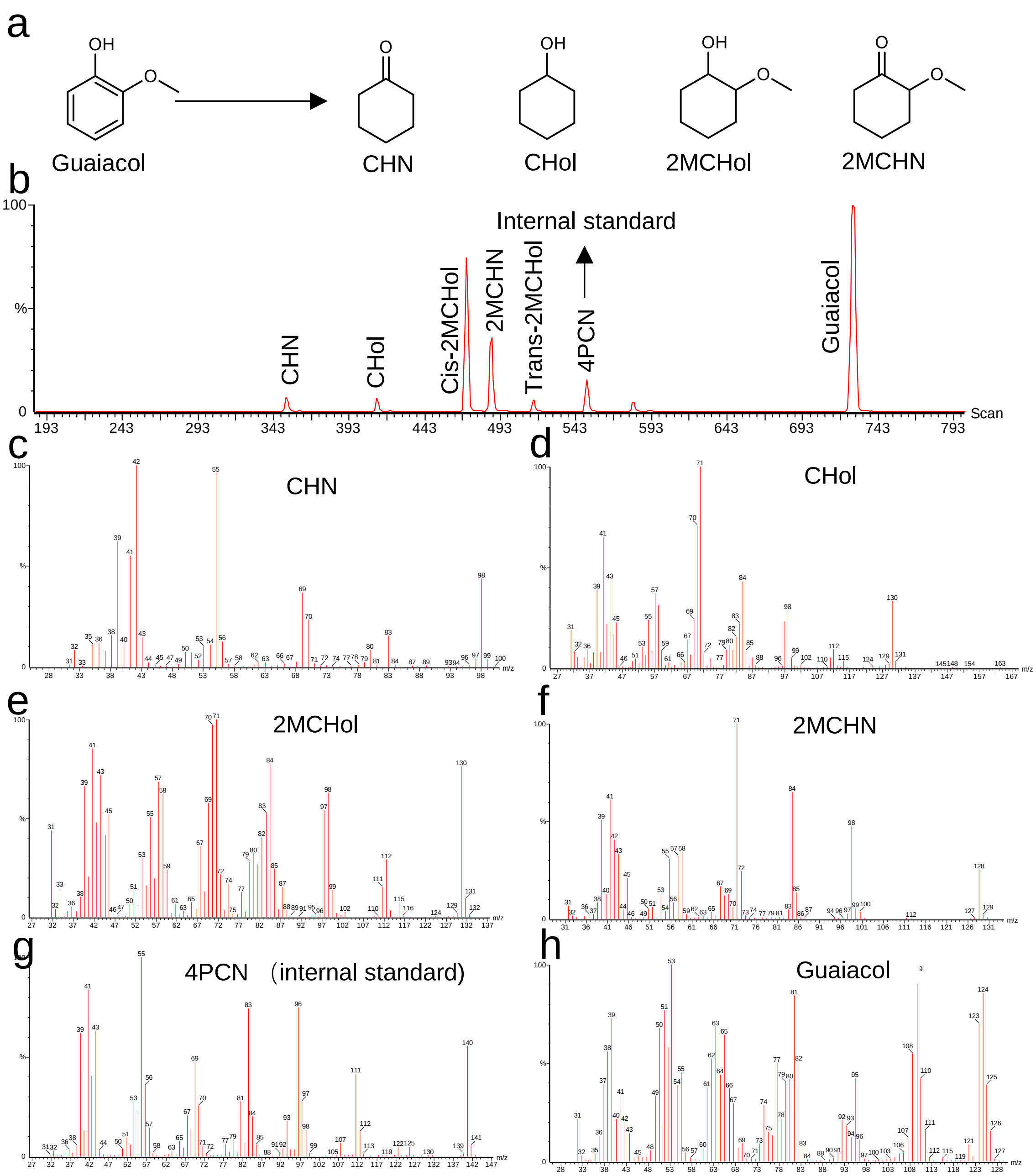

Figure S9. GC-MS results of products from ECH of guaiacol on Rh/CF catalyst. (a) ECH guaiacol leads to CHN, CHol, 2MCHol, and 2MCHN. (b) GC-MS analysis of ECH reaction. (c) Mass spectra from GC-MS analysis of CHN. (d) Mass spectra from GC-MS analysis of CHol. (e) Mass spectra from GC-MS analysis of 2MCHol. (f) Mass spectra from GC-MS analysis of 2MCHN. (g) Mass spectra from GC-MS analysis of 4PCN. (h) Mass spectra from GC-MS analysis of guaiacol.

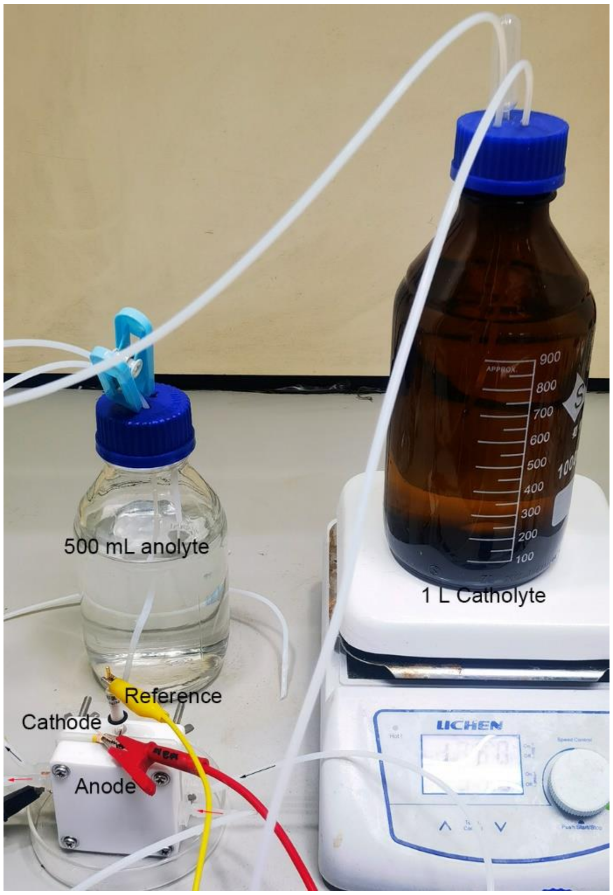

Figure S10. Stability test with 1 liter of 120 mM guaiacol solution at 300 mA/cm^2 for 32-h reaction.

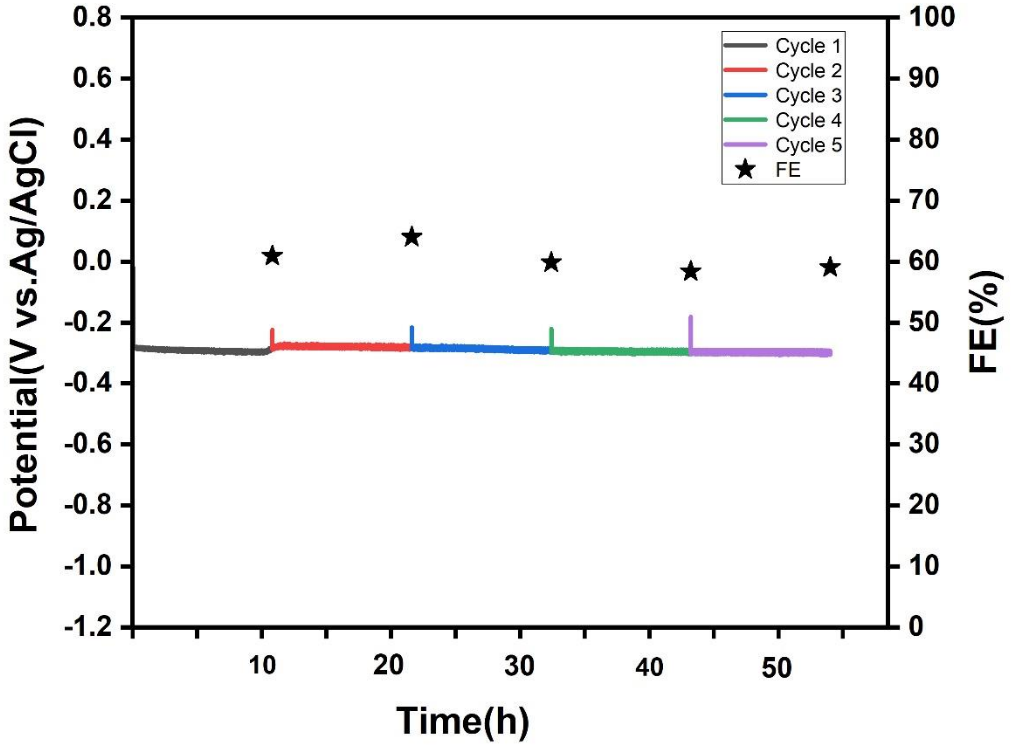

Figure S11. Cyclic stability test with 1 liter of 120 mM guaiacol solution ECH on Rh/CF catalyst using flow-cell system at 100 mA/cm^2. The cathodic potential is stable around -0.3 V and the FE of each cycle is stable at around 60% in a course of 5 cycles for a total of 60 hours.

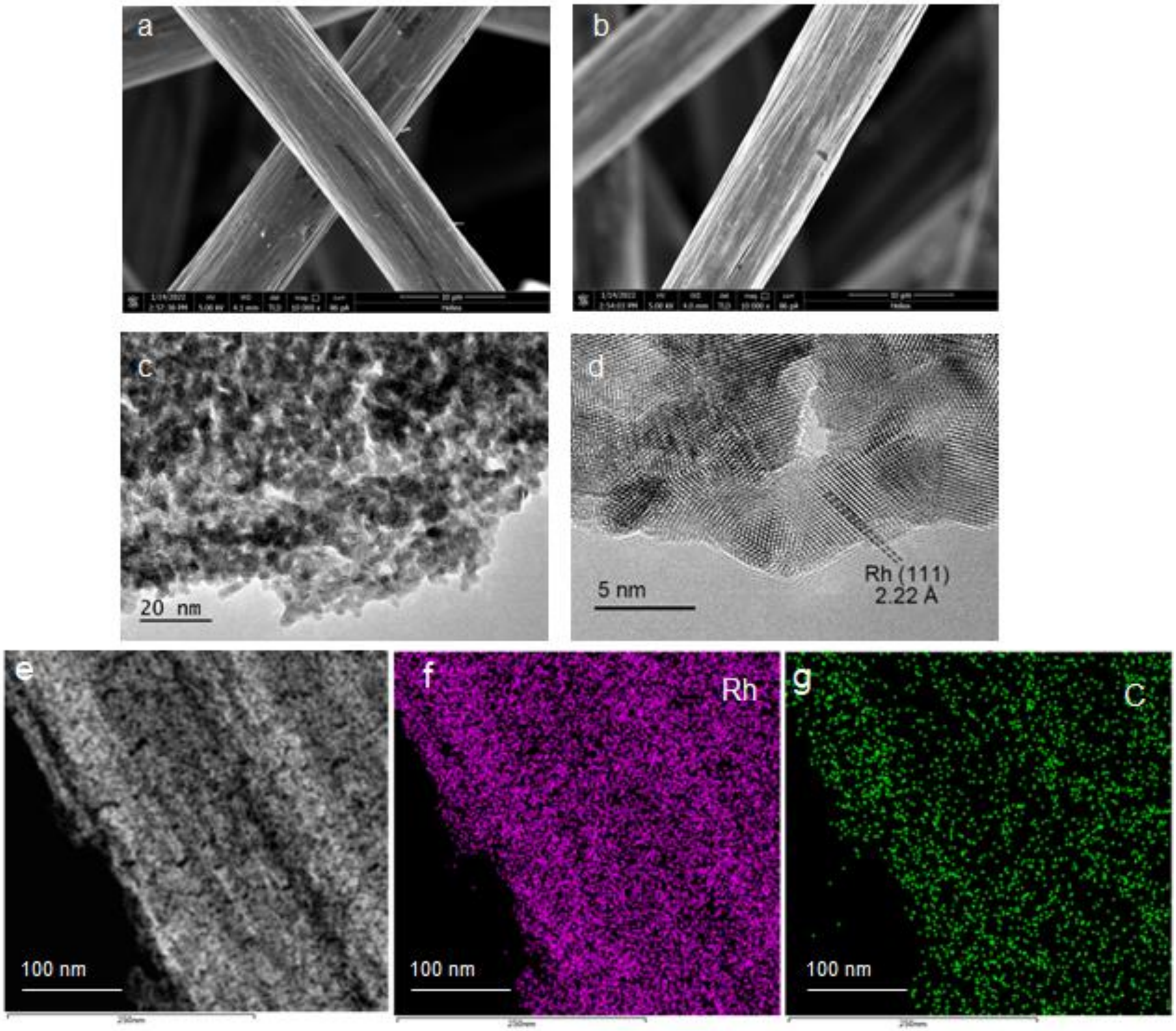

Figure S12. SEM (a-b) HRTEM (**c-d**), STEM (e), EDX mapping (**f-g**) of the Rh nanoparticles on carbon fiber after 32-h reaction, showing the uniform distribution of Rh on carbon fiber. Rh nanoparticles have diameter of ~5 nm with (111) crystal interplanar spacing of 2.22 Å. There is no apparent change of surface chemical composition after a course of 32-h ECH.

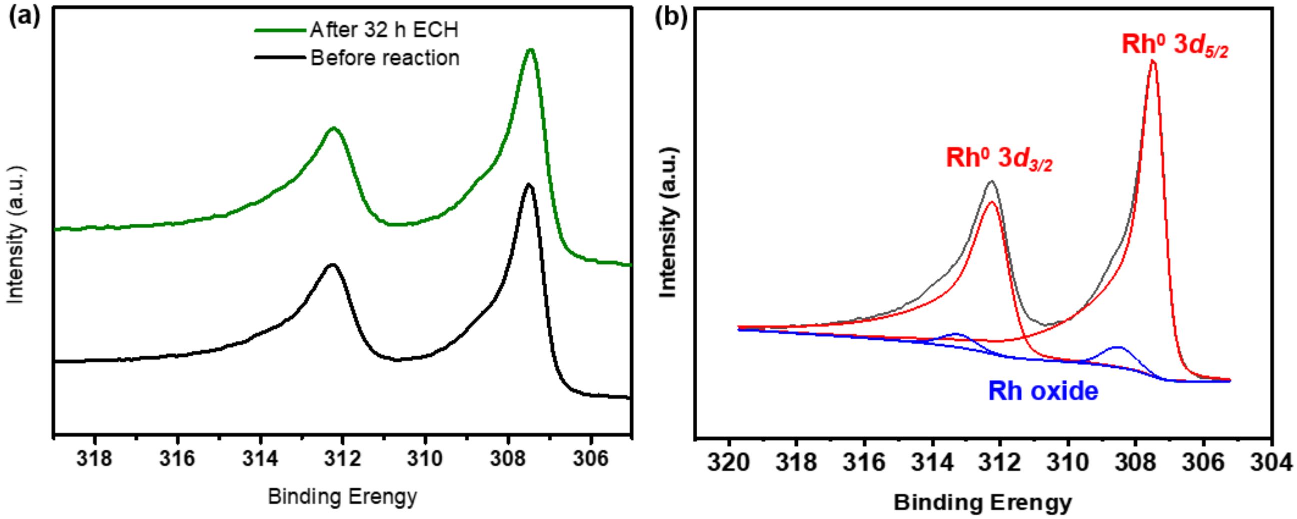

Figure S13. (a) XPS of Rh catalysts before and after 32-h reaction, showing no apparent change of surface chemical composition after ECH. (b) Fitting of Rh 3*d* before reaction, showing a small amount of Rh oxide (fitted spectra in blue) due to air oxidation.

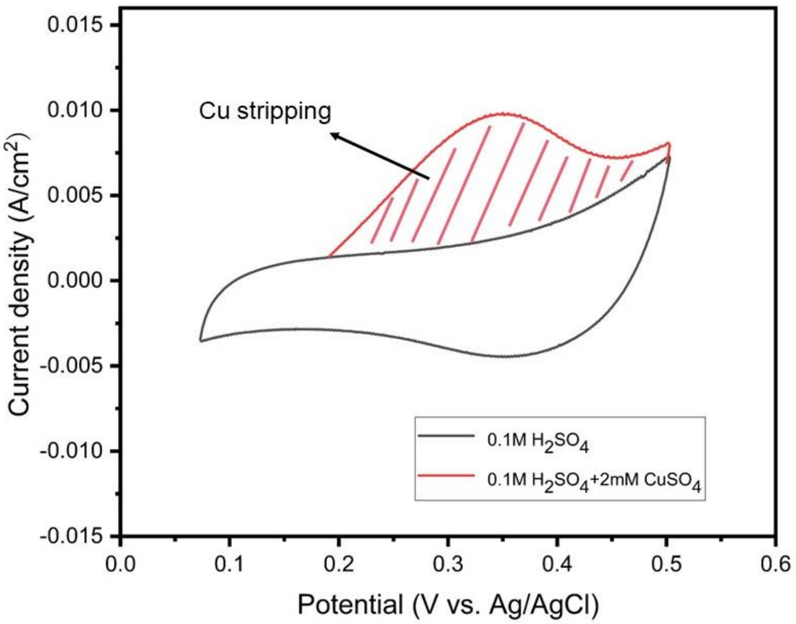

Figure S14. CV diagram for Cu_{UPD} of Cu on Rh/CF cathode.

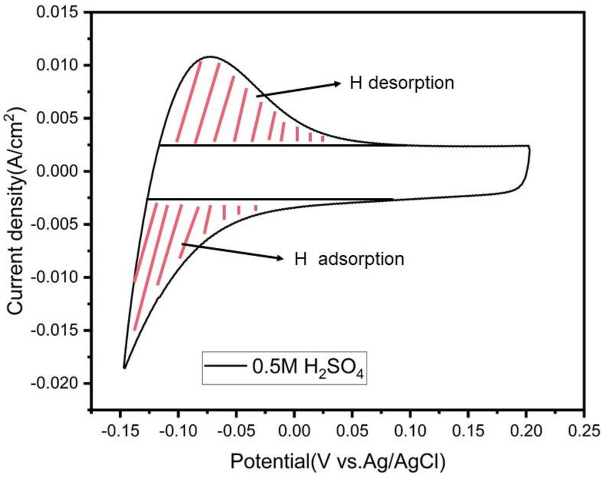

Figure S15. CV diagram for H_{UPD} of Rh/CF cathode.

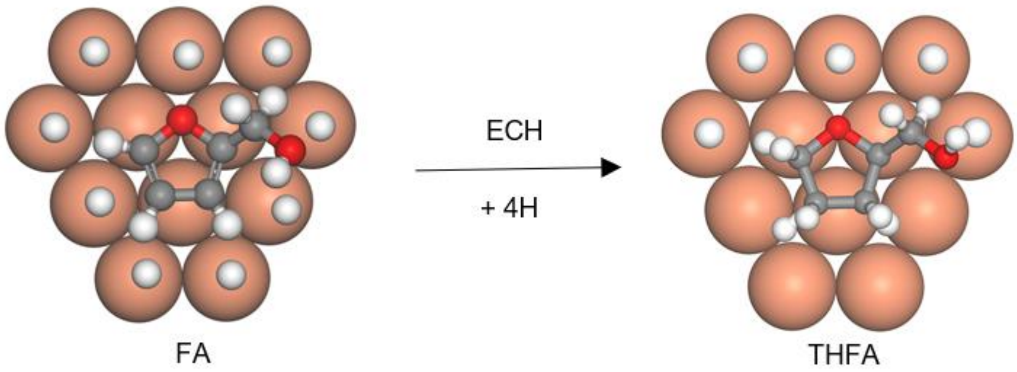

Figure S16. ECH of FA on Rh (111) crystal facet.

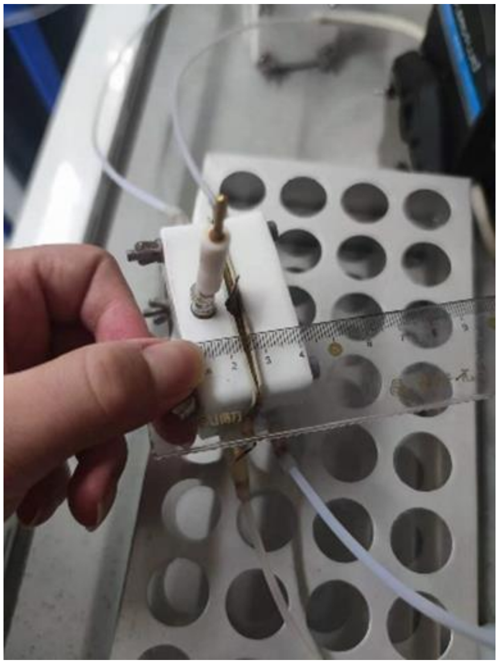

Figure S17. Flow-cell systems used in this study, showing the distance between anode and cathode.

References

1 Yang, F. *et al.* Rhodium phosphide: A new type of hydrogen oxidation reaction catalyst with non-linear correlated catalytic response to pH. *ChemElectroChem* **6**, 1990-1995, (2019).

2 Rand, D. A. J. & Woods, R. The nature of adsorbed oxygen on rhodium, palladium and gold electrodes. *Journal of Electroanalytical Chemistry and Interfacial Electrochemistry* **31**, 29-38, (1971).

3 Wei, C. *et al.* Approaches for measuring the surface areas of metal oxide electrocatalysts for determining their intrinsic electrocatalytic activity. *Chemical Society Reviews* **48**, 2518-2534, (2019).

4 Song, Y., Chia, S. H., Sanyal, U., Gutiérrez, O. Y. & Lercher, J. A. Integrated catalytic and electrocatalytic conversion of substituted phenols and diaryl ethers. *Journal of Catalysis* **344**, 263-272, (2016).

5 Liang, Z. *et al.* Direct 12-electron oxidation of ethanol on a ternary Au(core)-PtIr(shell) electrocatalyst. *Journal of the American Chemical Society* **141**, 9629-9636, (2019).

6 Zhou, Y. *et al.* Electrocatalytic upgrading of lignin-derived bio-oil based on surface-engineered PtNiB nanostructure. *Advanced Functional Materials* **29**, 1807651 (2019).

7 zhao, B., Chen, M., Guo, Q. & Fu, Y. Electrocatalytic hydrogenation of furfural to furfuryl alcohol using platinum supported on activated carbon fibers. *Electrochimica Acta* **135**, 139-146 (2014).

8 Green, S. K. *et al.* The electrocatalytic hydrogenation of furanic compounds in a continuous electrocatalytic membrane reactor. *Green Chemistry* **15**, 1869-1879 (2013).

9 Lam, C. H. *et al.* Electrocatalytic upgrading of model lignin monomers with earth abundant metal electrodes. *Green Chemistry* **17**, 601-609 (2015).

10 Li, Z. *et al.* Mild electrocatalytic hydrogenation and hydrodeoxygenation of bio-oil derived phenolic compounds using ruthenium supported on activated carbon cloth. *Green Chemistry* **14**, 2540-2549 (2012).

11 Garedew, M., Young-Farhat, D., Jackson, J. E. & Saffron, C. M. Electrocatalytic upgrading of phenolic compounds observed after lignin pyrolysis. *ACS Sustainable Chemistry & Engineering* **7**, 8375-8386 (2019).

12 Wijaya, Y. P., Smith, K. J., Kim, C. S. & Gyenge, E. L. Synergistic effects between electrocatalyst and electrolyte in the electrocatalytic reduction of lignin model compounds in a stirred slurry reactor. *Journal of Applied Electrochemistry* **51**, 51-63, (2021).

13 Wijaya, Y. P., Smith, K. J., Kim, C. S. & Gyenge, E. L. Hydrodeoxygenation of lignin related phenolic monomers in polar organic electrolyte via electrocatalysis in a stirred slurry catalytic reactor. *Green Chemistry* **24**, 7469-7480, (2022).

14 Wijaya, Y. P. *et al.* Electrocatalytic hydrogenation of guaiacol in diverse electrolytes using a stirred slurry reactor. *ChemSusChem* **13**, 629-639, (2020).

15 Wijaya, Y. P., Putra, R. D. D., Smith, K. J., Kim, C. S. & Gyenge, E. L. Guaiacol hydrogenation in methanesulfonic acid using a stirred slurry electrocatalytic reactor: Mass transport and reaction kinetics aspects. *ACS Sustainable Chemistry & Engineering* **9**, 13164-13175, (2021).

16 Andrews, E. *et al.* Performance of base and noble metals for electrocatalytic hydrogenation of bio-oil-derived oxygenated compounds. *ACS Sustainable Chemistry & Engineering* **8**, 4407-4418, (2020).

17 De Luna, P. *et al.* What would it take for renewably powered electrosynthesis to displace petrochemical processes? *Science* **364** (2019).

18 Leow, W. R. *et al.* Chloride-mediated selective electrosynthesis of ethylene and propylene oxides at high current density. *Science* **368**, 1228 (2020).

19 Jouny, M., Luc, W. & Jiao, F. General techno-economic analysis of CO_2 electrolysis systems. *Industrial & Engineering Chemistry Research* **57**, 2165-2177 (2018).

20 Yin, C. & Liu, G. Optimization of solvent and extractive distillation sequence considering its integration with reactor. *Processes* **9**, 565 (2021).
